# Tourism centres efficiency as spatial unites for applying blue economy approach: A case study of the Southern Red Sea region, Egypt

**DOI:** 10.1371/journal.pone.0268047

**Published:** 2022-07-27

**Authors:** Moaaz Kabil, Ebtehal Ahmed AbdAlmoity, Katalin Csobán, Lóránt Dénes Dávid

**Affiliations:** 1 Doctoral School of Economic and Regional Sciences, Hungarian University of Agriculture and Life Sciences (MATE), Godollo, Hungary; 2 Faculty of Urban and Regional Planning, Department of Regional Urban Development, Cairo University, Giza, Egypt; 3 Faculty of Economics and Business, University of Debrecen, Debrecen, Hungary; Tohoku University, JAPAN

## Abstract

This study aims to assess and analyse the efficiency of the tourism centres in the Southern Red Sea region, Egypt to apply coastal tourism development through the blue economy perspective. According to this aim, the study used two efficiency methods: Data Envelopment Analysis (DEA) and Free Disposal Hull (FDH). A total of 29 tourism centres were selected to conduct the DEA and FDH methods. These efficiency methods (DEA-FDH) used inputs and outputs variables to estimate the efficiency of the tourism centres. The selected inputs were the length of the shoreline (km), area (ha), tourism investments (million EGP), quality of coral reefs, numbers of hotels, and tourism accommodation capacity. While the outputs were employees’ number and tourists’ number. The results indicate that, generally, the tourism centres in the Southern Red Sea region of Egypt showed high-efficiency scores, which reflects their good preparedness to implement the various coastal tourism development strategies from the blue economy perspective. The tourism centres in the Safaga-Quseir tourism sector were the most efficient ones, regardless of the efficiency models used. While the tourist centres representing the Ras Banas tourism sector were the least efficient centres in the whole sample (29 tourism centres).

## Introduction

The recent global crises, especially the economic ones, have prompted the interest in searching for resilient development policies and paradigms that seek to achieve the sustainability conceptual kernel at the different economic, social, and environmental dimensions. One of the results of this interest in searching for those policies was the emergence of the concept of the blue economy [1,2]. Recently, precisely in 2012, the blue economy concept appeared on the global stage after the United Nations Conference on Sustainable Development (Rio +20/Earth Summit) that took place in Brazil [3]. The blue economy roots back to the continuous international community’s endeavour to implement the green economy approach at the international and regional levels [3,4]. After the global economic crisis in 2008, the international community and its institutional entities focused on activating mechanisms to boost the economic growth in a way that does not conflict with the environmental factors, and thus apply the idea of a green economy [5]. At the same time, the coastal states such as Small Island Developing States (SIDS) called for the necessity of paying attention to the role of oceans and their resources in enhancing economic growth. Hence was the actual appearance of the blue economy term for the first time in a report entitled "Green Economy in a Blue World" [3]. Therefore, it can be said that the blue economy conceptual kernel is similar, to some extent, to the green economy, with the difference in focusing on ocean resources and achieving optimal exploitation of them in consideration of sustainability principles. In conjunction with this momentum around the blue economy on the global stage, many different definitions of the blue economy emerged from several international and regional entities such as European Commission, United Nations Environment Program (UNEP), Organisation for Economic Co-operation and Development (OCED) and others. The main common idea for all these definitions was that the blue economy seeks to achieve human well-being and social justice through ocean-related economic activities and the diversity of existing marine ecosystems [3,6,7]. As a result of this diversity in the overall perspective of defining the blue economy, it consists of several economic sectors which collectively represent its comprehensive concept. These various economic blue economy sectors are classified into three main groups, namely: (a) Established Sectors: which have a long-term contribution to the blue economy such as aquaculture, fish processing industry, fisheries, shipbuilding & repair, water projects, coastal tourism, maritime transport, and marine extraction of oil and gas, (b) Emerging Sectors: which represent the sectors with high future potentials such as desalination, ocean energy, bioeconomy, offshore wind energy, and coastal & environmental protection, and (c) Enabler Sectors: such as shared infrastructure, sustainable use of the sea, maritime spatial planning, maritime security marine data, and common skills [3,6,8].

Among the various previous mentioned blue economy sectors, this study focused on the coastal tourism sector. Generally, the coastal tourism sector is one of the oldest, utmost importance, and fast-growing segments of the tourism industry [9,10]. Indeed, coastal tourism participated with a large proportion of the GDP of many countries, and it is expected that it alone will represent up to 26% of all ocean-related economic activities in the 2030 [11,12]. Additionally, compared to other blue economy sectors, the coastal tourism sector contributes about 83% of the gross value added (GVA) and it produces nearly 79% of the total blue economy job opportunities [6,13]. Furthermore, coastal tourism is a fragile and seasonal sector, which makes it vulnerable and sensitive to various environmental, social, and economic shocks [2]. For example, during the COVID-19 pandemic, the tourism industry was suffered from catastrophic economic losses as a consequence of the shutdown. According to the OCED, international tourism dropped by 80% in 2020 because of this crisis [14].

Here the blue economy plays an important role in enhancing and supporting the coastal tourism sector by focusing on achieving sustainability principles and increasing its resilience in facing various challenges such as climate change. The perspective of the coastal tourism sector in light of the blue economy approach was introduced through a set of key messages/principles such as financing sustainable coastal tourism strategies and actors, enhancing stakeholder participation in the coastal tourism development process, creating and growing new coastal tourism markets, protecting coastal areas and ecosystems from coastal tourism activities infrastructure, activating sustainability governance, harnessing the potential of urban communities and settlements to benefit from coastal tourism, enhancing the efficiency of coastal tourism resources, increase reliance on sustainable decision making in the coastal tourism sector, promoting the private sector role in coastal tourism, and decrease natural resources damage and the aesthetic value of these resources [9,10,13,15–19].

Despite the great paid international and regional attention to the blue economy as an approach seeking to achieve sustainable development using ocean resources, most of the academic production in this scientific area was merely initiatives and policies at the international or national levels, with limited strategies and implementation plans which effected on the true viability of applying the blue economy approach. Especially in the coastal tourism sector, which is the largest sector of the blue economy in size, but also the most affected sectors by the COVID economic crisis, and its recovery path is described as a “very lagged” [20].

Therefore, we need to overcome these barriers to witness the fruits of implementing the blue economy concept. Especially with the fact that coastal tourism appears as the considered the least blue economy sectors have been covered in articles and scientific researches, despite the vivid coastal tourism importance sharing of the gross value added (GVA) and providing job opportunities [21].

Consequently, several national, regional and global indicators have appeared to measure the blue economy approach to move towards the actual implementation of this approach into the practice [22]. These indicators were developed by various local and regional authorities and were based on different statistics and ways of calculation [23]. For example, Ocean Health Index (OHI) [24], European Commission blue economy reports [8,20], DG MARE’s initiative, maritime policy indicators by Eurostat, maritime economy statistics by Eurostat, French Marine Economic Data (FMED) [25], Ecorys [26], and others. These indicators can be divided into three main groups: (a) 1st group, indictors used the two basic blue economy measurements GVA and employment. (b) 2nd group, indicators focused on the economic and social impact of the blue economy such as investments, turnovers, revenues, or SMEs number, and (c) 3rd group, indicators used labour market characteristics to figure the blue economy size [27].

Despite the diversity of these measurements/indicators focusing on calculating the blue economy size, most of them applied on wide scales such as regional or international levels, with limited attempts to apply on local levels or at the economic sub-sectors of the blue economy such as coastal tourism sector. As well as the lack of transparency in both transferring the lessons learned from the measurement procedures in these indicators and the applicability of these indicators in different contexts and geographical areas [28]. Furthermore, these previous mentioned blue economy’s indicators aimed mainly to measure the size of the blue economy regardless of the readiness of the selected measured units (i.e., countries or regions) to implement the ideas of this development approach.

Accordingly, the main objective of this research paper is to understand the key messages of the blue economy for the coastal tourism sector and try to apply them into practice by measuring the readiness of the southern Red Sea region tourist centres in Egypt to apply the blue economy approach using DEA efficiency index. Thus, providing vivid helping tools for decision-makers to take the best developmental decisions to implement the blue economy concept at the local and regional levels.

In addition to, achieving the proposed research objective, this article seeks to assess three main hypotheses, as follows:

Hypothesis 1 (H1): “The principles of the blue economy can be better applied in untapped/raw coastal tourism centres rather than in the occupied ones."
Hypothesis 2 (H2): “The blue economy approach is applicable to apply at the local and regional levels as in the international one.”
Hypothesis 3 (H3): “The spatial developmental units (e.g. tourism centres) can be relied upon to apply the blue economy approach in the coastal tourism sector, as is the case in administrative units (e.g. governorates).”

The article is structured as follows. Section 2 presented the data and methodology. This section consisted of two main stages: the first stage is related to the spatial units whose efficiency will be measured to implement the blue economy concept (in this case, tourist centres), and the second stage is related to the selection of the calculation method of efficiency using Data Envelopment Analysis (DEA) method. The analysis and results are introduced in Section 3, followed by the discussion in section 4. Section 5 presented the conclusion of the study. Finally, the limitations of the study and future research directions are presented in Section 6.

## Materials and methods

### Study area

**Fig 1:**
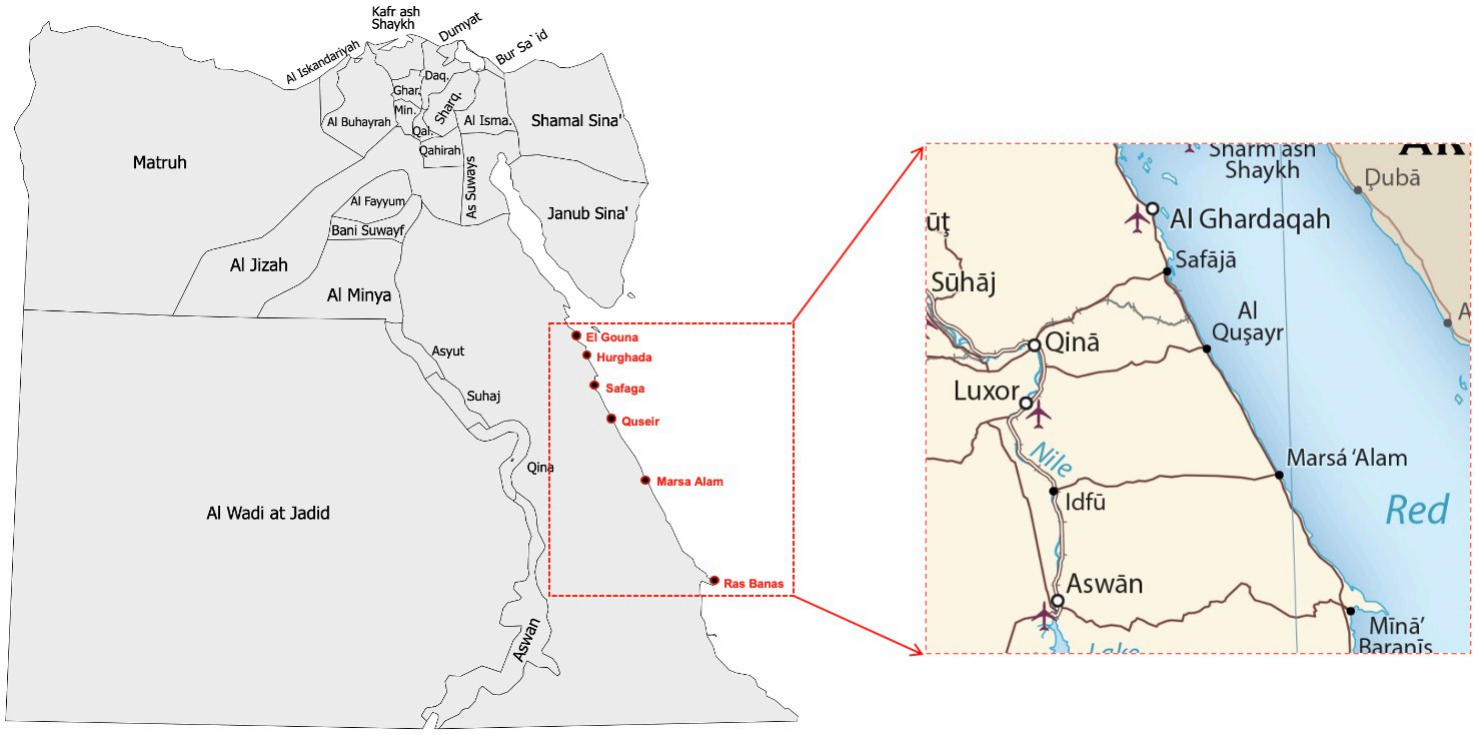
The southern Red Sea region, Egypt. Source: Adapted from [31].

This study discussed the efficiency of coastal tourism centres for applying the blue economy approach (coastal tourism sector) in the southern Red Sea region, Egypt. With its approximately 3000 km of coastline (Red Sea coast is 1850 km, and Mediterranean Sea coast is 1150 km) [29], the Egyptian coasts play an important role in the Egyptian economy in general and tourism development in particular. The Red Sea region is represented in the Red Sea Governorate which is a part of the southern Upper Egypt region (one of the seven planning regions in Egypt) [30]. The coast of the Red Sea region extends at a length of 1080 km, from the Gulf of Suez to the borders of Sudan. This study focused on the southern Red Sea region from Gouna in the north to Ras Benas in the south with a 575-km-long Red Sea coastline (Fig 1).

The choice of the southern Red Sea region as a study area for implementing the blue economy concept in the coastal tourism sector at the regional level was based on several reasons. The southern Red Sea region has unique tourism developmental resources such as coral reefs, mangroves, and other different environmental sea ecosystems that host thousands of marine species [31]. Additionally, the Red Sea region generally is considered a premier tourism destination visited by about 1.2 million tourists annually, bringing around $1.2 billion foreign currency, and providing nearly 275,000 job opportunities [32]. Furthermore, at the governmental and official level, the Egyptian government set various major environmental policies, programs, and initiatives in cooperation with many international bodies such as USAID, to enhance the achievement of the sustainable development goals (SDGs) in the economic development of the Red Sea region [32], paving the way for realizing the various principles of the blue economy approach. Also, in recent years, the Red Sea region attracted several individuals or institutional investors in different economic sectors and one of them is the coastal tourism [33]. Additionally, this region includes many urban communities and cities such as Hurghada, Safaga, and Quseir, which have an urban structure that allows them to play a major role in leading the tourism development process and ensuring its sustainability [34]. Finally, the Red Sea region faced various challenges that always require searching for sustainable development approaches such as the blue economy, and the most important of these challenges was the presence of many untapped coastal areas rich in resources, pollution resulting from infrastructure and tourism services, coastal habitats loss, and the destruction of coral reefs [33,35].

For all these pervious mentioned reasons, the southern Red Sea region is considered a suitable environment for applying the ideas and principles of the blue economy, especially in the coastal tourism field.

### Research method

The research method is divided into two main stages: (I) Dividing the southern Red Sea region into spatial tourism units and (II) Efficiency benchmarking of tourism performance in the southern Red Sea region. These two stages are presented as follows.

#### Stage (I): Dividing the Southern Red Sea region into spatial tourism unites

According to the main objective of this paper, it relied on a frontier statistical method to measure the efficiency of tourism performance of the Southern Red Sea Region in Egypt. Before discussing the proposed method, it is necessary to understand and define the spatial units on which the efficiency method will be applied. Despite the debate about the possibility of measuring the blue economy at the national, regional, or local levels, this research does not aim “directly” to measure the blue economy (give a precise value) but rather aims to know the adequacy of coastal tourism spatial units in applying the blue economy approach ideas and key messages.

Although there were administrative boundaries that divide the urban geographic areas into different units, this article will not consider it as the main boundaries for dividing the Southern Red Sea Region into spatial tourism development units for many reasons. For example, these administrative boundaries do not take into account the spatial distribution of development resources, but rather they are boundaries that serve and support political and administrative targets at the national, governmental, and institutional levels. Additionally, the coastal tourism sector is a fragile, seasonal, and resilient one, which makes it vulnerable to various changes and it is necessary to rely on boundaries targeting realistic and developmental bases far from the administrative oriented ones. Also, these administrative boundaries do not consider the presence of sufficient infrastructure for developing the tourism sector. Finally, the main goal of this research focuses on coastal tourism development, so it needs a specific spatial division targeting the tourism sector, instead of the administrative boundaries that consider all dimensions and economic sectors such as industry, trade, agriculture, politics, governance, and others.

**Fig 2:**
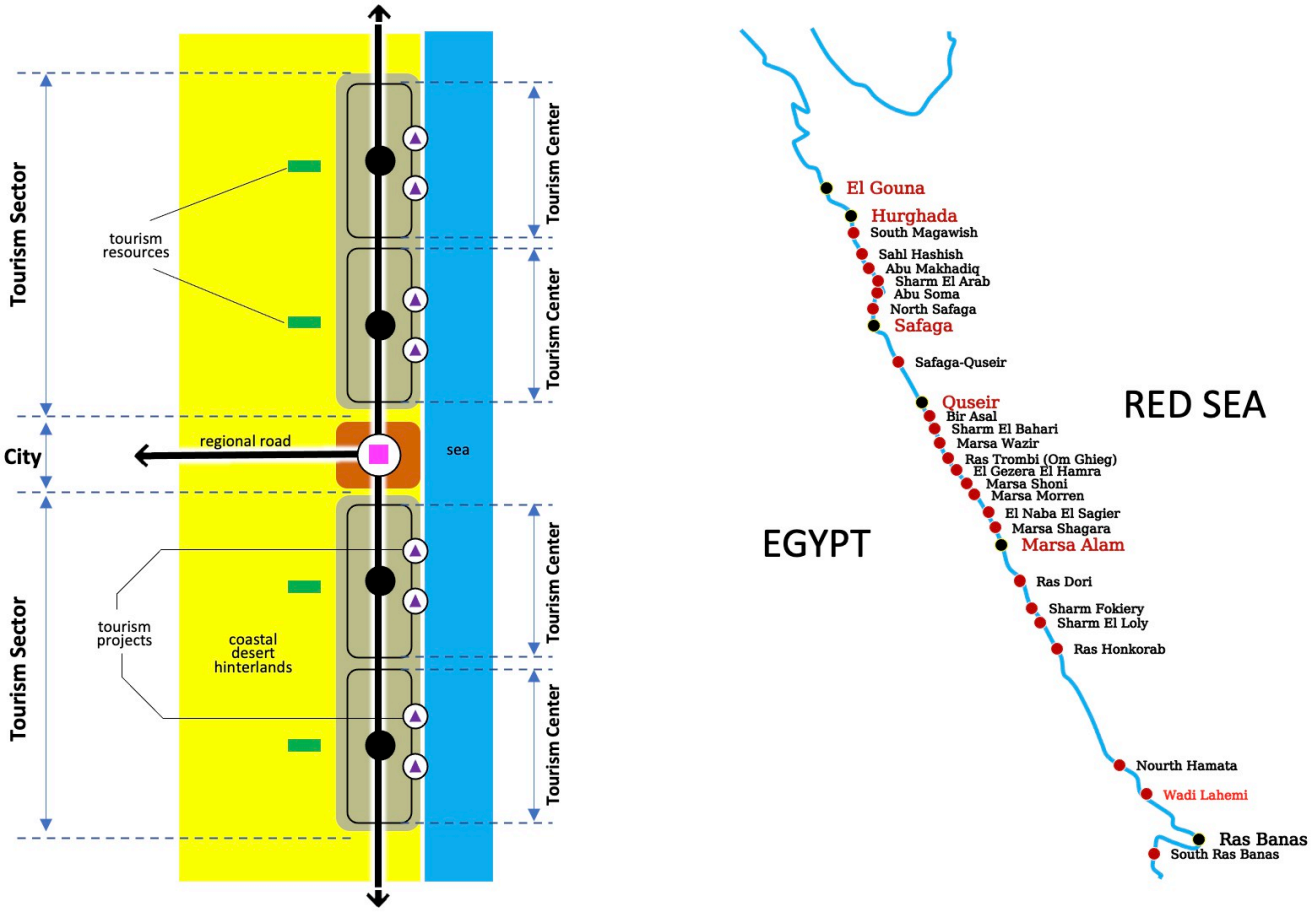
Tourism centres establish idea. Source: Researcher based on [36].

Accordingly, the southern Red Sea region will be divided into spatial Tourism Development Centres (TDC), and then the efficiency of applying the blue economy conceptual kernels in these spatial tourism centres will be measured. Tourism Development Center (TDC) is a spatial geographical area that consists of a combination of several tourism projects such as tourist villages, hotels, tourist resorts, public or private beaches, restaurants, coastal tourist activities centres (recreational or sports), and other various tourism resources. The lands of these projects are allocated through the Egyptian Tourism Authority (ETA), which owns the proposed coastal land acquisition for tourism development. Fig 2 shows the idea of creating and devising the spatial tourism development centres.

It is also worth noting that these spatial divisions (tourism centres) for the southern Red Sea region have been established in a study published by the Japan International Cooperation Agency (JICA), the Egyptian Tourism Development Authority (TDA), and the Egyptian Ministry of Tourism (MOT), entitled “The study on tourism development projects in the Arab Republic of Egypt”, in 2000 [36]. But the divisions of the tourism centres in this research were different from their counterpart in the previously mentioned study, in that coastal cities have been considered as one of the tourism centres because of their potential, resources, and development capabilities that can push the process of tourism development and achieve the blue economy ideas.

According to the Egyptian Ministry of Tourism (MOT), dividing the coastal regions into spatial tourism development centres has many goals such as opening new tourism markets, increasing tourism growth, treating the decline of the traditional tourist destinations, preserving environmental sensitivity of reserved areas, maximizing the benefit of tourism investments, and linking tourism development with urban growth to overcome the regional disparities in the Egyptian urban system. Additionally, the division of the southern Red Sea region into tourist centres depends on several criteria, such as the threshold distance, geographical characteristics of the coast, natural or man-made physical barriers, regional land uses, urban settlements distribution, accessibility and mobility, characteristics of urban communities, elements of tourist attractions, and potentials of the tourism region hinterlands.

#### Stage (II): Efficiency benchmarking of tourism performance in the southern Red Sea region

Generally, efficiency is the concept of comparing the productivity of economic units (e.g., firms) against each other, so it is classified as a comparative analysis. The efficiency analysis between different units should be at the same level of inputs and outputs factors. In recent decades, techniques and tools for assessing efficiency have greatly developed, producing massive academic literature in various sciences and fields, including the tourism one. For example [37–46], used different efficiency models in the tourism sector to identify the best practice units and then develop plans and strategies to raise the efficiency of the least efficient units.

**Fig 3:**
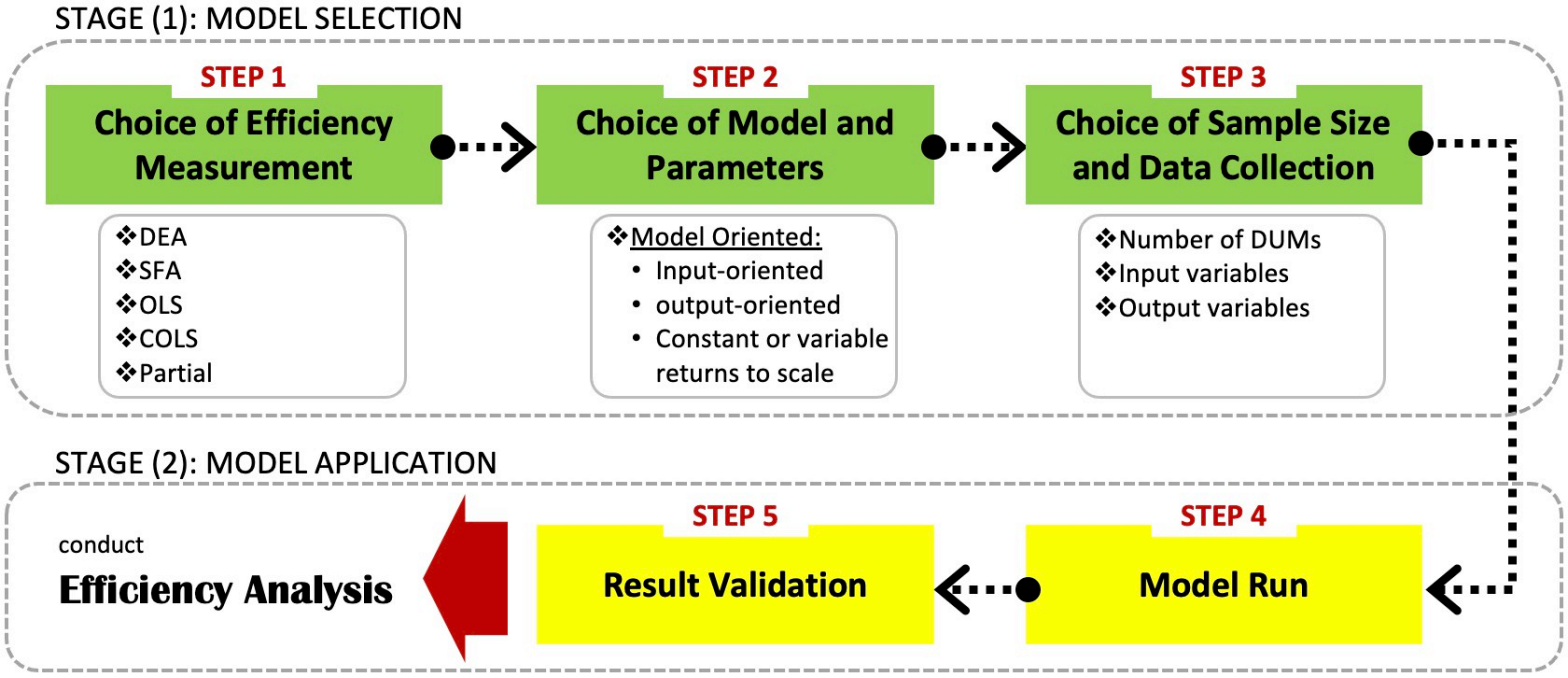
Efficiency assessment analysis procedures.

The process of conducting the efficiency benchmarking analysis goes through main two stages: model selection and model application. Regarding the model selection stage, there are three main steps: choice appropriate efficiency measurement approach, choice model parameters, choice sample size, and collecting data. Followed by the model application stage designed for running the selected model and validating the findings. Each step has its unique sequent elements, Fig 3 depicts the benchmarking process for applying efficiency assessment analysis in our research.

To measure the efficiency of the tourism centres in the southern Red Sea region for applying the blue economy approach, the researchers used two frontier methods: Data Envelopment Analysis (DEA) and Free Disposal Hull (FDH). Regarding Data Envelopment Analysis (DEA) method, the research adopted two input-oriented models namely: the variable returns to scale (VRS) and the constant returns to scale (CRS). The choice of DEA as the main analysis method in this study was based on serval reasons such as DEA’s ability to handle multiple inputs and outputs simultaneously, do not need restrictive functional equations, it can divide efficiency into several elements, it is a unit invariant (do not need standardization), and it is useful for target setting which fits with the goal of the search and the collected data [47–49].

Data Envelopment Analysis (DEA) is a non-parametric envelopment frontier method, was initially propounded by the three professors Abraham Charnes, William Cooper, and Edwardo Rhodes (CCR) in 1978 [50], and extended by Banker, Charnes, and Cooper (BCC) in 1984 [51]. DEA is a linear programming frontier optimization approach. The conceptual kernel of DEA is to estimate the relative efficiency of homogenized decision-making units, known as DMUs, that use multiple inputs which represented the resources and produce some outputs [52]. Efficiency using DEA is represented with a single number (*θ*) on a scale from zero to 1, with the rule that (0 < *θ* ≤ 1), where the value of 1.0 represent efficient DMU [53]. The efficiency or inefficiency of a particular DMU is assessed by the distance of how far this DMU is from the constructed frontier [54]. Also, it is worth mentioning that the efficiency numbers it’s not on a linear scale, so it cannot be said that the DMU with a 0.5 efficiency score is half as efficient as one that’s at 1.0. Additionally, DEA not only calculates DMU’s efficiency, but also identifies the inefficiency causes and then ranks the DMUs according to their efficiency, as well as pinpoints how to improve the efficiency of these inefficient units.

DEA could be applied using two models: input-oriented or output-oriented. The input-oriented model focused on minimizing inputs by keeping the outputs constant, while the output-oriented model focused on maximizing the outputs by keeping the inputs without changing. In this research, the input-oriented model is used to estimate the efficiency of coastal tourism centres for applying the blue economy approach. This choice was based on the target of this study and the collected data, where the inputs were the most controllable factors. To estimate the efficiency score for the coastal tourism centres using the input-oriented model, the study opted for the two original DEA concepts: constant return to scale (CRS) and variable return to scale (VRS). CRS assumption was introduced by Charnes, Cooper, and Rhodes, so it is known as (DEA-CCR) [50]. However, the VRS assumption is known as DEA-BCC for Banker, Charnes, and Cooper [51].

The Free Disposal Hull (FDH) method is also a non-parametric analysis to estimate the efficiency of DMUs. It is developed by Deprins, Simar, and Tulkens in 1984 [55], and extended by Lovell in 1993 [56]. The FDH concept considers any DMU as an efficient unit when there are no other units that produce outputs more than or equal to it. Additionally, FDH focused on the relationship between inputs and outputs based on what is known as the free disposability [57]. The main advantage of FDH compared to the two DEA concepts (CCR & BCC) is that it does not require a convexity [58]. Thus, in this study, the researchers considered FDH as a second used technique to measure the efficiency of the coastal tourism centres in applying the blue economy.

Using these two models, DEA and FDH, to measure the efficiency of the tourism centres in the southern Red Sea region was based on five main reasons. Firstly, the differences in the assumptions of these two methods, especially the “convex & nonconvex”, was a motive to use the two methods simultaneously. DEA models are convex ones. In other words, the targets corresponded to theoretical DMUs (unreal DMUs), which may be meaningless in real-life practices such as the evaluation of the tourism centre’s efficiency in this research. On the other hand, the FDH model is a nonconvex one. Thus, targets are compared with more real-life’s DMUs. In some practices, the observed DMUs are more meaningful when compared to real DMUs than virtual ones [59]. Consequently, running the two models in this research is more appropriate and helps to understand the real reasons behind the efficiency scores. Secondly, running DEA and FDH in parallel was recommended by many researchers who conducted the efficiency analysis in their articles [60,61]. Some researchers have even gone further by inventing a new method that mixed the DEA and FDH models, which reflects the strong relationship between these two models [62]. Thirdly, although DEA and FDH share the same mathematical programming structure, DEA is formulated as a series of linear programs, while FDH is formulated as a series of mixed-integer programs [56]. Because of this difference, FDH is frequently used as a second method with DEA as it helps to ease the computational burden of solving DEA problems. Fourthly, using two efficiency assessment models, especially when the dataset is small, is considered extremely valuable and increase the robustness and credibility of the final findings. Finally, Finally, most of the scholarly literature—that concerned with conducting efficiency analysis—used the two methods simultaneously such as [63,64].

The linear programming problems for the three efficiency measurements in this study (DEA-CCR, DEA-BCC, and FDH) could be described as follows:

$${min}_{\phi ,\delta }\phi$$ (1)

$$s.t.\;\phi x_{s}-X\delta \geq 0$$ (2)

$$Y\delta \geq y_{s}$$ (3)

δ≥0(DEA-CCR) (4)

eδ=1(DEA-BCC) (5)

$$\delta_{s}\in \left\{0,1\right\}\;\;(FDH)$$ (6)

Where (S) represented the number of tourism centres that used (*x*_*s*_) inputs to produce (*y*_*s*_) outputs. The data matrices are formed by (*x*_*s*_) and (*y*_*s*_) for X-axis and Y-axis, respectively. Additionally, (*δ*) represented the non-negative vector that formed the linear combinations of the (S) tourism centres. Finally, (*e*) represented the suitably dimensioned vectors. For estimating the efficiency results of the DEA-CCR model, Problems (1) to (4) were solved. While for the DEA-BCC model, Problems (1) to (5) were solved together. Finally, Problems (1) to (4) and (6) were solved to estimate the efficiency score of the FDH model. Additional details about these previous non-linear programming problems can be found in [51,58].

To calculate the efficiency measurements in this paper, the Benchmarking package from the R programing language was used [59]. Additionally, the results were visualized by using the novel web interactive interface (deaR-Shiny), which was devised by Benitez, Coll-Serrano, and Bolós in 2021 [60].

## Results and discussion

In this study, as mentioned previously, the DEA and FDH will be used to estimate the efficiency of tourism centres in the Egyptian Southern Red Sea region for applying the blue economy conceptual kernel, especially in the coastal tourism sector. These analytical methods use several inputs and outputs to compare the efficiency of different DMUs (in this case, tourism centres). The used inputs for each tourism centre were the length of the shoreline (km), area (ha), tourism investments (million EGP), quality of coral reefs, numbers of hotels, and tourism accommodation capacity. While the outputs were employees’ number and tourists’ number. This data was collected from different Egyptian official sources such as the strategic plan of the Red Sea, investment opportunities report in the Red Sea governorate, annual report of tourism statistics, economic census, and the Egyptian Tourism Authority (ETA). Due to the unavailability of precise data of some elements, these elements data collected at the level of tourism sectors and not tourism centres such as the quality of coral reefs.

Concerning the rules of conducting DEA, the inputs and outputs in this study were selected according to two main guidelines. First, inputs and outputs should be consequential, functional, comprehensive, and positive. Second, the relationship between the number of observations (DMUs/coastal tourism centre) and the number of variables (inputs and outputs) must follow at least one of these three rules: *s* ≥ 2(*x*+*y*), *s* ≥ 3(*x*+*y*), or *s* ≥ 2*x*y. Where *s*, *x*, and *y* represented the number of DMUs, inputs, and outputs, respectively [61–64]. In our dataset, all these three rules were applied, where (*s* = 29, *x* = 6, and *y* = 2). The following Table 1 shows the descriptive statistics for the different used inputs and outputs in our study.

**Table 1:** Descriptive statistics of coastal tourism centres data (inputs & outputs) of DEA.

No.	Variable	Obs ^a^	Measurement unit	Minimum	Maximum	Mean	^b^ Std.
**INPUTS**							
**1**	**Length of Shoreline**	29	km	2.21	73.07	19.78	15.17
**2**	**Area**	29	ha	136	3843300	180943.3	725457.5
**3**	**Investments**	29	Million EGP	7	4473	725.91	1000.03
**4**	**Quality of Coral Reefs**	29	Scale (1–10)	4	10	7.9	1.8
**5**	**Numbers Hotels**	29	Hotel	0	67	5.24	12.74
**6**	**Accommodation Capacity**	29	Room	1828	69510	26786.41	21291.69
**OUTPUTS**							
**7**	**Employees Numbers**	29	Employee	1428	58096	10709.33	11494.81
**8**	**Tourist Numbers**	29	Tourist	0	600000	112481.3	143682.9

^a^ Obs: Represent the number of observations (coastal tourism centres),

^b^ Std: Represent the standard deviation.

The following Table 2 shows the results of running different models of DEA. In addition to the previous three mentioned DEA models (DEA-CCR, DEA-BCC, and FDH), this study calculated the average of them (AVG) and conducted the scale efficiency measurement (SE). The scale efficiency measurement can be obtained by (SE = DEA-CCR / DEA-BCC).

**Table 2:** DEA for measuring the efficiency of the coastal tourism centres in the Southern Red Sea region, Egypt.

No.	DMUs (Tourism Centers)	Tourism Sector	DEA-CCR	DEA-BCC	FDH	^a^ AVG	^b^ SE
**1**	**Gouna**	Hurghada-Safaga	**1.00**	**1.00**	**1.00**	**1.00**	**1.00**
**2**	**Hurghada**		**1.00**	**1.00**	**1.00**	**1.00**	**1.00**
**3**	**South Magawish**		**1.00**	**1.00**	**1.00**	**1.00**	**1.00**
**4**	**Sahl Hashish**		0.98	0.99	1.00	0.99	0.99
**5**	**Abu Makhadiq**		**1.00**	**1.00**	**1.00**	**1.00**	**1.00**
**6**	**Sharm El Arab**		0.50	0.87	0.99	0.79	0.57
**7**	**Abu Soma**		**1.00**	**1.00**	**1.00**	**1.00**	**1.00**
**8**	**North Safaga**		**1.00**	**1.00**	**1.00**	**1.00**	**1.00**
**9**	**Safaga**	Safaga-Quseir	**1.00**	**1.00**	**1.00**	**1.00**	**1.00**
**10**	**Safaga-Quseir**		**1.00**	**1.00**	**1.00**	**1.00**	**1.00**
**11**	**Quseir**		**1.00**	**1.00**	**1.00**	**1.00**	**1.00**
**12**	**Bir Asal**	Quseir-Marsa Alam	0.93	1.00	1.00	0.98	0.93
**13**	**Sharm El Bahari**		**1.00**	**1.00**	**1.00**	**1.00**	**1.00**
**14**	**Marsa Wazir**		0.57	1.00	1.00	0.86	0.57
**15**	**Ras Trombi (Om Ghieg)**		**1.00**	**1.00**	**1.00**	**1.00**	**1.00**
**16**	**El Gezera El Hamra**		**1.00**	**1.00**	**1.00**	**1.00**	**1.00**
**17**	**Marsa Shoni**		**1.00**	**1.00**	**1.00**	**1.00**	**1.00**
**18**	**Marsa Morren**		**1.00**	**1.00**	**1.00**	**1.00**	**1.00**
**19**	**El Naba El Sagier**		**1.00**	**1.00**	**1.00**	**1.00**	**1.00**
**20**	**Marsa Shagara**		0.99	1.00	1.00	1.00	0.99
**21**	**Marsa Alam**	Marsa Alam-Ras Benas	0.96	0.98	0.99	0.98	0.98
**22**	**Ras Dori**		**1.00**	**1.00**	**1.00**	**1.00**	**1.00**
**23**	**Sharm Fokiery**		**1.00**	**1.00**	**1.00**	**1.00**	**1.00**
**24**	**Sharm El Loly**		**1.00**	**1.00**	**1.00**	**1.00**	**1.00**
**25**	**Ras Honkorab**		0.78	0.99	1.00	0.93	0.79
**26**	**North Hamata**		**1.00**	**1.00**	**1.00**	**1.00**	**1.00**
**27**	**Wadi Lahemi**		**1.00**	**1.00**	**1.00**	**1.00**	**1.00**
**28**	**Ras Benas**	Ras Benas	0.57	0.82	0.89	0.76	0.70
**29**	**South Ras Benas**		0.19	1.00	1.00	0.73	0.19
**Average**			**0.91**	**0.99**	**1.00**	**0.97**	**0.92**

Bold results represent the efficiency of tourism centres in the whole used DEA models.

^a^ AVG: Represent the average of the three DEA models,

^b^ SE: Represent scale efficiency (DEA-CCR / DEA-BCC).

The averages of all the different used or calculated efficiency measurements are presented high performance, and it can be ranked FDH, BCC, AVG, SE, CCR with averages of 1, 0.99, 0.97, 0.92 and 0.91, respectively.

Considering the perspective of the different used DEA models in this study, the DEA-CCR results show that 20 out of 29 tourism centres were efficient, with a percentage of up to 69%. The most inefficient tourism centres were South Ras Benas, Sharm El Arab, and Ras Benas with CCR efficiency scores 0.19, 0.5, and 0.57, respectively. While the other inefficient DMUs were not far away from being efficient with scores between 0.78 to 0.99. Similarly, the results of the DEA-BCC model, where about 83% (24 out of 29) of the tourist centres were efficient. All inefficient tourism centres in this test were as close as possible to efficiency by achieving scores ranging from 0.82 to 0.99, so it can be said that most, if not all, tourist centres according to this test are considered “figurative” efficient. This BCC model findings also reveal that most of the efficient tourism centres in the BCC were recorded as efficient ones in the CCR model except for Bir Asal, Marsa Wazir, Marsa Shagara, and South Ras Benas. This confirmed the readiness of these tourism centres to apply the concept of coastal tourism development according to the blue economy approach. Moving to FDH model results, all the tourism centres were efficient except for Ras Benas with a 0.89 efficiency score.

Regarding the scale efficiency measure (SE), 20 of 29 tourism centres score 1.00 to describe as efficient units. Except for the Safaga-Quseir spatial tourism sector, all other tourism sectors have some inefficient tourism centres, noting that the Ras Benas tourism sector is still an inefficient one, where its two belongs to tourist centres (Ras Benas & South Ras Benas) scored 0.7 and 0.19 in SE measure, respectively.

**Fig 4:**
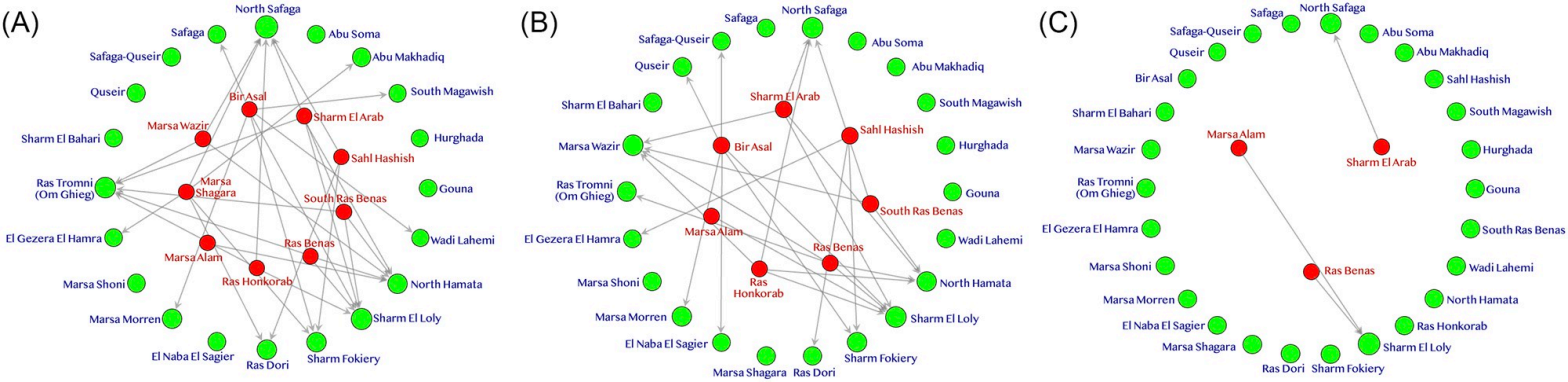
The reference plot for DEA-CCR, DEA-BCC, and FDH. A. The graphical results of the DEA-CCR model. B. The graphical results of the DEA-BCC model. C. The graphical results of the FDH model.

Fig 4 shows the graphical results of the different tourism centres’ efficiency scores depending on the used model. This figure is known as a reference plot, and it is extracted by using the deaR-shiny web interface [60]. This figure is formed of three sub-figures (Fig 4A–4C) that represented the results of DEA-CCR, DEA-BCC, and FDH analysis models, respectively. In these Figures, the efficient DMUs represented by green circles, while the inefficient ones have a red colour. The size of the circle in this plot expressed the total number of obtained lambdas/intensities according to the DMU’s reference sets. Additionally, the grey arrows represented the link between the inefficient DMUs and their nearest homogenizing reference set.

It is worth noting that all the tourist centres represented in urban cities (Gouna, Hurghada, Safaga, and Quseir) were efficient in the whole conducted except Marsa Alam city. This is considered an important indicator for the ability of these urban communities to play significant roles and lead the coastal tourism development process in preparation for the implementation of the blue economy concept.

It is clear from the previous results of the DEA models that most of the tourism centres in the Southern Red Sea region in Egypt have achieved high levels of efficiency for applying the blue economy approach in general and in the coastal tourism sector in particular. This is due to various reasons, such as the diversity of the different tourism development resources in this coastal strip. Additionally, the high accessibility of these spatial tourism centres with the major urban cities in the Southern Red Sea region such as Hurghada, Safaga, El Quseir, and Marsa Alam, which increase the prosperity of these tourism centres and their ability to attract numerous local and international tourists. Moreover, some of these coastal cities are considered international tourism destinations, such as Hurghada, which has the best diving areas because of its diverse coral reefs. Finally, some statistics confirmed this distinction for the tourism centres in the Red Sea Region, where, according to the Egyptian Ministry of Tourism (MOT), the Red Sea Region accounts for about 31% of international tourism in Egypt.

One of the other reasons that increase the ability of the tourism centres in the Southern Red Sea region to apply the blue economy approach, is the near location of these tourism centres to the Nile Valley, especially Luxor and Aswan cities. This easy mobility with a network of regional roads paved the way to organize tourism trips for these two cities (one day trip), which will enrich the Red Sea region’s tourists experience. The Southern Red Sea region is also characterized by the presence of international airports such as Hurghada Airport and Marsa Alam Airport, which enhances the concept of the efficiency of the tourism centres in this region and its ability to attract international tourists and provide numerous job opportunities for the local communities.

Despite the low-efficiency scores in some tourism centres in the Southern Red Sea region, this is because these areas are untapped coastal areas and have not been developed at present. For example, the Ras Banas tourism sector achieved a low-efficiency record in all the used efficiency models in this study, but this coastal area is rich in natural tourism resources such as coral reefs, diving areas, sandy beaches, mild weather, and others.

The high values of efficiency models applied to the tourism centres in the Egyptian Southern Red Sea region for developing the coastal tourism through the blue economy perspective, open the way for decision-makers to make development decisions that support these advantages and increase the optimal utilization and development of coastal resources. Especially considering Egypt’s limited participation in international agendas and conferences concerned with the blue economy. Where Egypt participated only in the "Sustainable Blue Economy Conference”, which was hosted by the Kenyan capital "Nairobi" at the end of November 2018 (from November 26 to 28), and focused on new technologies and innovation in the seas, oceans, rivers and lakes, to explore challenges, opportunities and possible partnerships [65]. This study and its results are considered a realistic look at the ability of the Egyptian coastal regions to adapt to the principles of the blue economy, especially in a country with 3000 km of coastline between the Red Sea and the Mediterranean.

Regarding the assessment of the three research hypotheses, the first hypothesis (H1) which claimed the preference of the untapped tourist centres compared to the occupied ones in applying the blue economy principles, was rejected. Since most of the inefficient tourism centres were untapped ones such as Ras Benas and Ras Honkorab. In addition, the research findings confirmed the second hypothesis (H2), which claimed the applicability of applying the blue economy approach in the local and regional practices. By testing the local tourism centres in a national region (Red Sea Region) for applying the blue economy principles, the results revealed the existence of some efficient and inefficient tourism centres, which may help decision-makers to take appropriate development decisions when applying this approach in coastal areas in Egypt. Similarly, hypothesis 3 (H3) was confirmed by the research findings. Whereas, tourism centres, as spatial development units, appeared more convenient for applying the blue economy principles compared to the administrative units, despite the difficulty of collecting data from these tourism centres.

## Conclusions

This study conducted an efficiency analysis of 29 tourism centres in the southern Red Sea region, Egypt to identify their readiness to implement the blue economy approach in the coastal tourism sector. Two different efficiency indicators were selected: DEA (CCR-BCC) and FDH. Before starting to test the efficiency of the proposed spatial tourism units, the southern Red Sea region coast was divided into spatial units (tourist centres) depending on the availability of tourism resources in each centre. The input variables were the length of the shoreline, area, tourism investments, quality of coral reefs, numbers of hotels, and tourism accommodation capacity, whilst the outputs were employees’ number and tourists’ number. In general, the results of the selected efficiency analysis models revealed the high readiness of most of the tourism centres in the Southern Egyptian Red Sea region (except for the Ras Banas tourism sector) to implement and develop coastal tourism from the perspective of the blue economy approach. The results also showed the importance of urban cities in leading the future coastal tourism development process in the region due to the high levels of efficiency achieved by these cities.

Additionally, this study highlighted the importance of focusing on the precise determining of the readiness of the different units to apply the blue economy approach in its various economic sectors. This importance of identifying the unites ready to implement the blue economy principles (regions or countries), should be associated with the current great development in the search for statistical indicators measuring the blue economy at the various regional, national and international levels. Therefore, a better future vision will be devised about how to take advantage of the various blue economy initiatives and implement them into practice, thus achieving the developmental and practical dimension of the various blue economy sectors.

## Limitations and future research

This study is not exempt from limitations, through which future research can be carried out. One of these key limitations is the lack of information. In the coastal tourism field, various criteria can be considered to develop this sector (e.g., tourism services capacity, tourism spending rates, % of local and international tourists, and others). The choice of tourism centres (not official administrative boundaries) as the main spatial unities for the efficiency analysis, case this lack of information. However, it is worth mentioning that this choice of tourism centres as spatial units to measure efficiency had many justifications, which were mentioned previously in the research. It is also possible to collect these indicators through questionnaires and surveys, which may give more robust results in the future.

Regarding future research, many ideas that seek to study any of the blue economy sectors at the regional and local levels can emerge from the main idea of this research paper. For example, identifying the ability and efficiency of coastal ports to support the coastal tourism development process from the perspective of the blue economy, and others.
